# Dielectric Barrier Discharge Ionization Mechanisms: Polycyclic Aromatic Hydrocarbons as a Case of Study

**DOI:** 10.1021/acs.analchem.2c03279

**Published:** 2022-12-20

**Authors:** Marcos Bouza, Julio García-Martínez, Bienvenida Gilbert-López, Sebastian Brandt, Juan F. García-Reyes, Antonio Molina-Díaz, Joachim Franzke

**Affiliations:** † Analytical Chemistry Research Group, Department of Physical and Analytical Chemistry, University of Jaén, Campus Las Lagunillas, 23071Jaén, Spain; ‡ ISAS—Leibniz Institut für Analytische Wissenschaften, Bunsen-Kirchhoff-Str. 11, 44139Dortmund, Germany

## Abstract

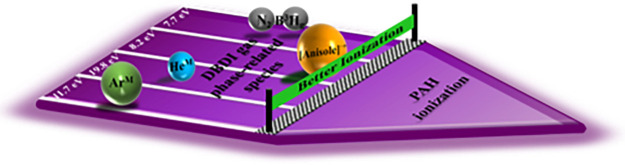

Dielectric barrier discharge ionization (DBDI) is a versatile tool for small-molecule mass spectrometry applications, helping cover from polar to low polar molecules. However, the plasma gas-phase interactions are highly complex and have been scarcely investigated. The ionization mechanisms of plasmas have long been assumed to be somewhat similar to atmospheric pressure chemical ionization (APCI). Here, we evaluated the ionization mechanisms of a two-ring DBDI ion source, using different discharge gases to analyze vaporized liquid samples. Polycyclic aromatic hydrocarbons (PAHs) were used as model analytes to assess the mechanisms’ dominance: protonation, [M + H]^+^, or radical ion species formation, [M]^·+^. In the present work, two different ionization trends were observed for APCI and DBDI during the PAH analysis; the compounds with proton affinities (PA) over 856 kJ/mol were detected as [M + H]^+^ when APCI was used as ionization source. Meanwhile, independently of the PA, DBDI showed the prevalence of charge exchange reactions. The addition of dopants in the gas-phase region shifted the ionization mechanisms toward charge exchange reactions, facilitating the formation of [M]^·+^ ion species, showing anisole a significant boost of the PAH radical ion species signals, over nine times for Ar-Prop-DBDI analysis. The presence of high-energy metastable atoms (e.g., He^M^) with high ionization potentials (IE = 19.80 eV) did not show boosted PAH abundances or extensive molecule fragmentation. Moreover, other species in the plasma jet region with closer and more appropriate IE, such as N_2_ B^3^Π_g_ excited molecules, are likely responsible for PAH Penning ionization.

## Introduction

Ambient ionization mass spectrometry (AIMS) has evolved and matured during the last decade. Notably, the simplicity, easy operation, speed, and specificity of AIMS ion sources enabled a wide variety of applications.^1,2^ Among the several approaches, plasma-based ion sources are one of the most important ones in AIMS.^3−5^ The nature of the discharge is influenced by different factors, but mostly the power supply (direct-current, alternative-current (AC), or microwave) and the selected discharge gas define the plasma discharge behavior.^6,7^ The design versatility and the expanded chemical coverage granted by plasma-based ion sources, enabling ionization of both polar and low polar species, are one of their unique desirable features.^8,9^ One of the more versatile and flexible approaches in plasma-based devices are those based on dielectric barrier discharges (DBDs).^10,11^ It allows multiple electrode-dielectric configurations using AC power supplies to generate the discharge.^12^ Low-temperature plasma (LTP),^13^ dielectric barrier discharge ionization (DBDI),^14,15^ and active capillary plasma ionization (ACaPI)^16^ are among the most representative DBD-based ion sources. The applicability of DBD has been reported in fields ranging from environmental science,^17,18^ home security,^8,19^ bioanalytics,^20^ to forensics,^21^ probing DBD’s relevance. Still, fundamental studies of DBD ionization mechanisms are scarce. Plasma-based ion sources are assumed to rely on similar ionization mechanisms as atmospheric pressure chemical ionization (APCI).^22^ However, the nature of the discharge, the presence of a discharge gas, and the discharge microenvironment influence and conform a unique gas-phase chemistry.^23,24^ ACaPI is one of the DBD-like ion sources where these parameters have been thoroughly evaluated. Parameters such as the discharge gas,^25^ the effect of the humidity,^26^ and the use of charge exchange promoting molecules^27^ were evaluated to understand ACaPI behavior. A better understanding of fundamental aspects during the ionization may help to enhance the performance of the different applications for these ion sources.

**Figure 1:**
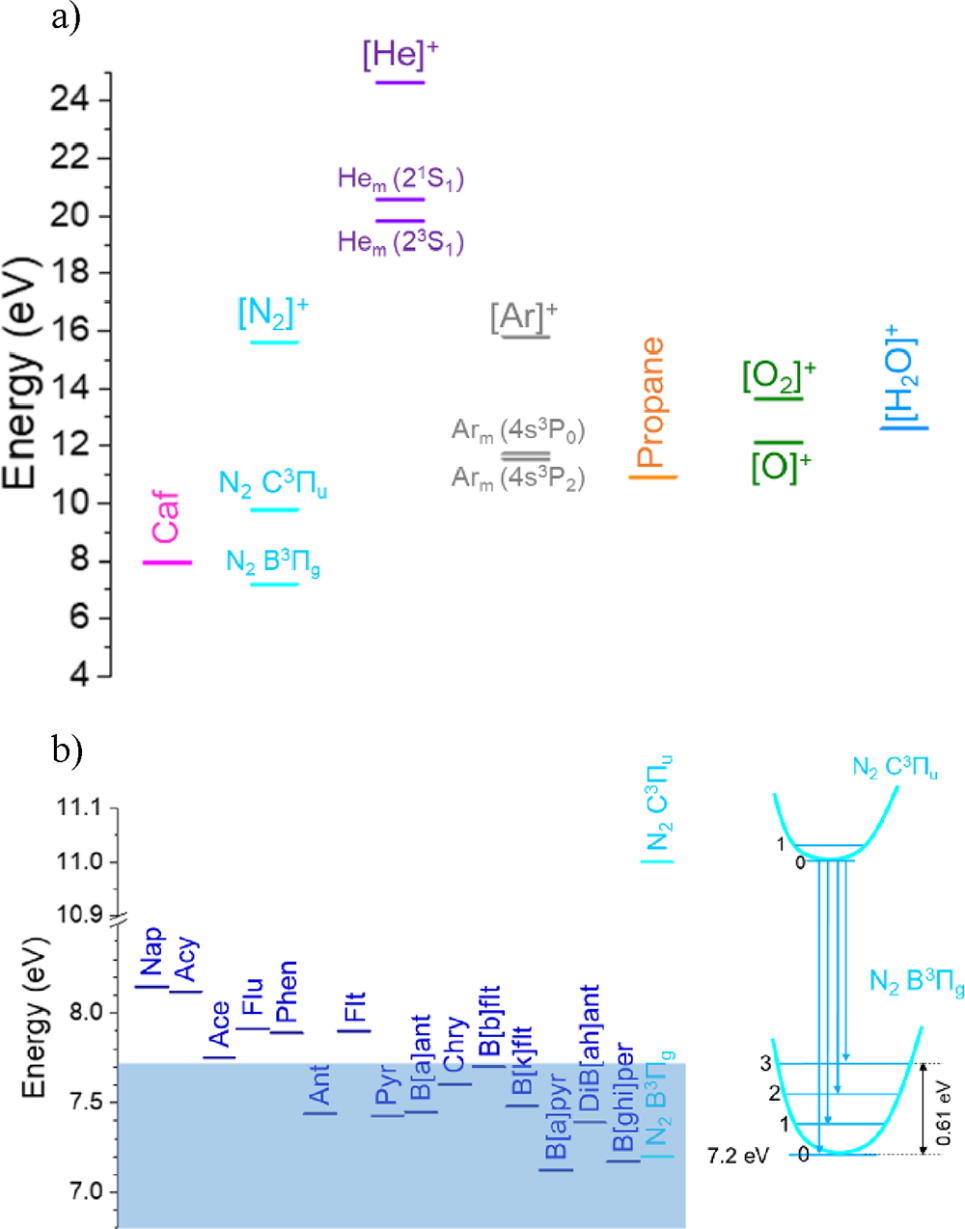
Diagram of the ionization and excitation potential for different species present in the DBDI plasma evaluated. (a) Ionization and excitation potentials for the main species that contribute to the plasma operation and maintaining, and the target analytes evaluated. (b) PAH ionization potential with respect to excited N_2_ C^3^Π_u_ and N_2_ B^3^Π_g_. The blue-shadowed region represents the PAH potentially ionized via Penning ionization with excited N_2_; zoom-in on the energy of the different states of excited N_2_ is also collected. Caf states for caffeine.

Here, we proposed a thorough evaluation of DBDI gas-phase chemistry coupled to liquid chromatography (LC)-MS and compared it to the commercial APCI ion source, a corona discharge. To further evaluate the gas-phase chemistry, different discharge gases, helium, and an argon–propane mixture will be used to connect the plasma ionization to key analyte properties such as ionization potential (IE) (see Figure 1) or proton affinities (PA). Polycyclic aromatic hydrocarbons (PAHs) were selected to understand the predominance of proton transfer or charge exchange mechanisms during plasma operation. PAHs were also chosen because of their structural similarities and the important differences in IE or PA (see Table S1). Different dopants were injected post-column to shift and control the ionization mechanisms. This article reports on the unique gas-phase chemistry observed during LC-DBDI-MS and the possibility to control and tailor the ionization mechanisms to promote a more favorable formation of radical ions, important for analyses of PAHs, alkanes, and other low polar molecules sometimes hard to detect using plasma-based ionization sources.

## Experimental Section

### Materials

An analytical standard containing a mixture of 16 PAHs (TraceCERT PAH Calibration Mix) in acetonitrile (ACN) at 10 mg/L was purchased from Sigma-Aldrich (St. Louis, USA). The mixture contains naphthalene (Nap), acenaphthylene (Acy), acenaphthene (Ace), fluorene (Flu), phenanthrene (Phen), anthracene (Ant), fluoranthene (Flt), pyrene (Pyr), benzo[*a*]anthracene (B[*a*]ant), chrysene (Chry), benzo[*b*]fluoranthene (B[*b*]flt), benzo[*k*]fluoranthene (B[*k*]flt), benzo[*a*]pyrene (B[*a*]pyr), dibenz[*a*,*h*]anthracene (DiB[*ah*]ant), benzo(*g*,*h*,*i*)perylene (B[*ghi*]per), and indeno[1,2,3-*cd*]pyrene (In[123 *cd*]py). This mixture was diluted at 5 mg/L for the analysis using a mixture of 1:1 water/ACN as solvent. Chlorobenzene (CB), fluorobenzene (FB), toluene (Tol), anisole (Ani), B[*b*]flt, and B[*a*]pyr were obtained from Sigma-Aldrich (St. Louis, USA) and Fluka (Buchs, Switzerland). For LC/MS analysis, LC–MS-grade water, ACN, and formic acid were from Sigma-Aldrich (St. Louis, USA).

### DBDI and APCI Analyses

The DBDI source used is a ring-to-ring DBD consisting of a glass capillary with two annular electrodes separated by 10 mm, as previously described.^28^ The DBD plasma is generated between the two rings and inside of the capillary when 2.5 kV is applied to the front electrode, using an in-house-built high-voltage square wave generator at 20 kHz, while 150 mL/min of inert gas is flowing through the capillary. Helium (purity 99.999%) and argon–propane (Ar-Prop) were used to evaluate the effect of the discharge gas; a premixed bottle of argon (purity 99.999%) containing 3000 ppm of propane was bought from Air Liquide (Madrid, Spain).

The DBD ion source was adapted to fit into a commercial APCI housing (Agilent Technologies, Santa Clara, USA) so that comparable results can be attained with the commercial APCI ion source. The positioning of the discharge probe has been optimized previously to favor proper nebulization and vaporization of the LC eluent.^29^ The current supplied to the APCI was 4 μA.

### LC and Direct Infusion MS

The separation of the analytes was done in an ultra-high-pressure liquid chromatography (UHPLC) system, Agilent 1290 Infinity UHPLC (Agilent Technologies, Santa Clara, USA). Ten microliters of the PAH mixture was injected. The mobile phases used were ultrapure water with 0.1% of formic acid (A) and ACN with 0.1% of formic acid (B). The presence/absence of the formic acid effect was evaluated during the present work, but as observed before, formic acid had negligible influence during PAH ionization.^30^ The separation was carried out using a reversed-phase C18 column (ZORBAX Eclipse Plus C18 RRHD 2.1 × 100 mm, 1.8 μm, Agilent Technologies, Santa Clara, USA). The chromatographic method held the initial mobile phase composition (50% B) constant for 1 min, followed by a linear gradient up to 85% B at 3 min. After that, it was increased to 100% B at 10 min and held at 100% B for 2 min. The flow rate was set at 200 μL/min to favor a rapid separation.

Individual analyses of the selected analytes were done to corroborate experimental results. B[*b*]flt and B[*a*]pyr calibrations were carried out by direct injection using a column gap. The same mobile phases as previously described were used. Ten microliters of the evaluated analytes was injected. A flow rate of 200 μL/min of a 50/50 mixture of mobile phases A and B was used.

The detection was achieved using a time-of-flight (TOF) mass spectrometer (Agilent 6220 Accurate-Mass TOF, Agilent Technologies, Santa Clara, USA) in positive ion mode. The parameters were held constant for DBDI and APCI to avoid potential ionization variations due to the vaporization or ion transfer conditions: vaporizer temperature, 300 °C; nebulizer, 15 psi; capillary voltage, 3.5 kV; drying gas flow, 3.4 L/min at 350 °C; fragmentor, 175 V; skimmer, 65 V; octopole RF, 250 V. The acquisition range was from *m*/*z* 50 to *m*/*z* 1100 at a scan rate of 1.42 spectra/s. The instrument was operated in the 4 GHz high-resolution mode. Agilent MassHunter Data Acquisition software (version B.04.00) was used to acquire the data, and the results were processed with Agilent MassHunter Qualitative Analysis software (version B.04.00) for individual compound analyses. PAH-targeted analyses were done using Skyline.^31^

### Use of Dopants

A syringe pump (74,900 series, Cole-Parmer Instruments, Illinois, USA) was used to infuse the dopants. The compounds were added post-column using a PEEK T-piece. The dopant flow rate was typically 20 μL/min, representing ca. 10% of the LC effluent.

## Results and Discussion

### APCI vs He-DBDI

**Figure 2:**
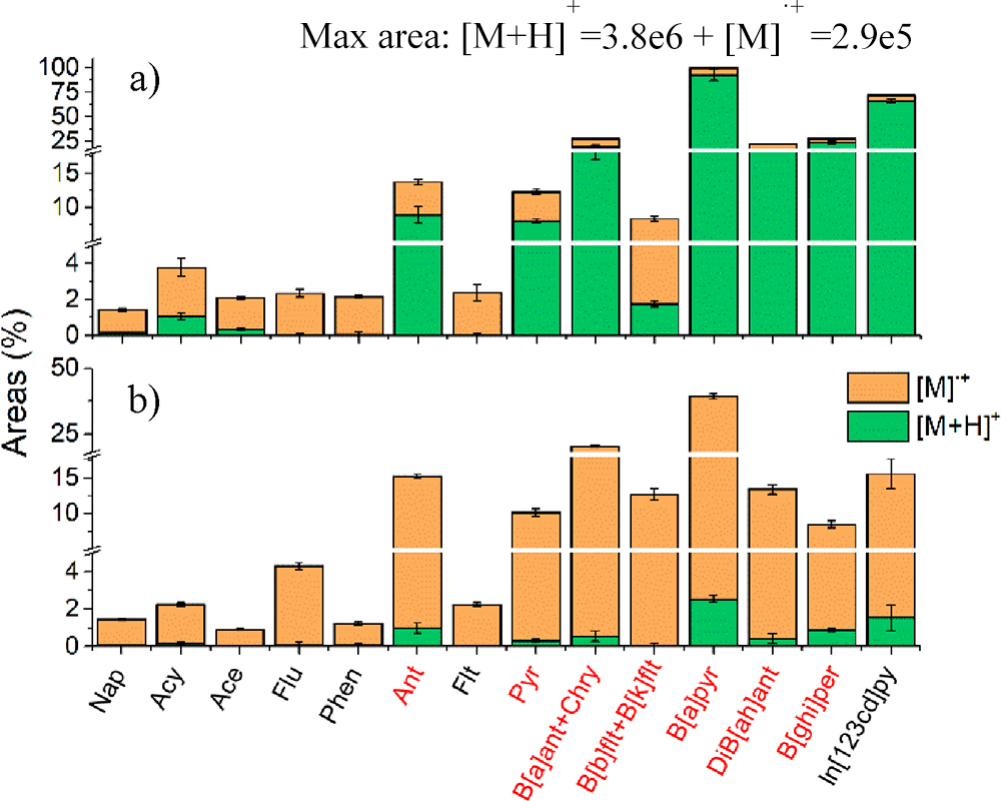
Areas of [M]^·+^ and [M + H]^+^ signals for the PAHs under study. (a) APCI; (b) He-DBDI. The analyses were relativized to the sum of the areas of [M + H]^+^ and [M]^·+^ signals of benzo[*a*]pyrene detected with APCI. The radical ion species are represented in green and the protonated ion species in orange. Highlighted in red are the compounds with IE <7.70 eV.

Different target molecules, discharge gases, and post-column dopants were proposed to understand the ionization mechanisms of DBDI. The ionization of He-DBDI was compared to APCI (Figure 2). As observed in Figure 2a, 7 out of 16 PAHs showed a favorable formation of [M + H]^+^ ions for APCI analysis, with PAHs having PA ≥856 kJ/mol (see Table S1). On the other hand, radical ion formation was the dominant ion species type for He-DBDI (Figure 2b), likely ionized through charge exchange reactions.^18^ Even in the case of DBDI, PAHs with high PA (i.e., Ant or B[*a*]pyr) favored the partial formation of [M + H]^+^; the protonated ions for Ant and B[*a*]pyr represented 6.5% of the total observed signal. These data indicate two different ruling ionization mechanisms for two plasma-based ion sources.

The following reactions are assumed as the main ruling mechanisms during APCI*:
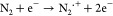
1
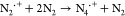
2
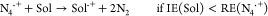
3
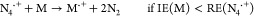
4
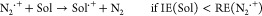
5
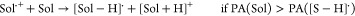
6
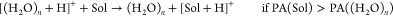
7
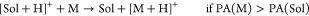
8
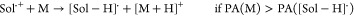
9
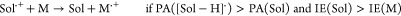
10
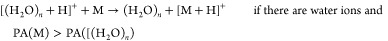
11
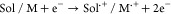
12

*The reactions have been adapted from refs (32, 24). RE means recombination energy.^33^

APCI and DBDI were operated in an ambient atmosphere, specifically in a N_2_-rich ionization environment used as vaporization gas. The cascade of reactions that starts the corona discharge and sustains the plasma requires high-energy electrons and N_2_ (eq 1), followed by a third-body collision to generate N_4_^·+^ ions (eq 2). The presence of N_4_^·+^ during APCI analysis will suggest the likelihood of the accepted gas-phase reactions. The three approaches, APCI, He-DBDI, and Ar-DBDI, showed a signal at *m*/*z* 56.0113, highly likely attributed to N_4_^·+^ with a relative mass error of −7.1 ppm. We observed favored radical ion species for low PA compounds during APCI analysis (Figure 2a). Both N_4_^·+^ and N_2_^·+^ promoted charge exchange reactions (eqs 3, 4, and 5). The reaction criterion was matched or overcome for all the molecules (solvents and analytes) used in the present work. The formation of radical ion species when a noble discharge gas is used will be discussed later.

**Figure 3:**
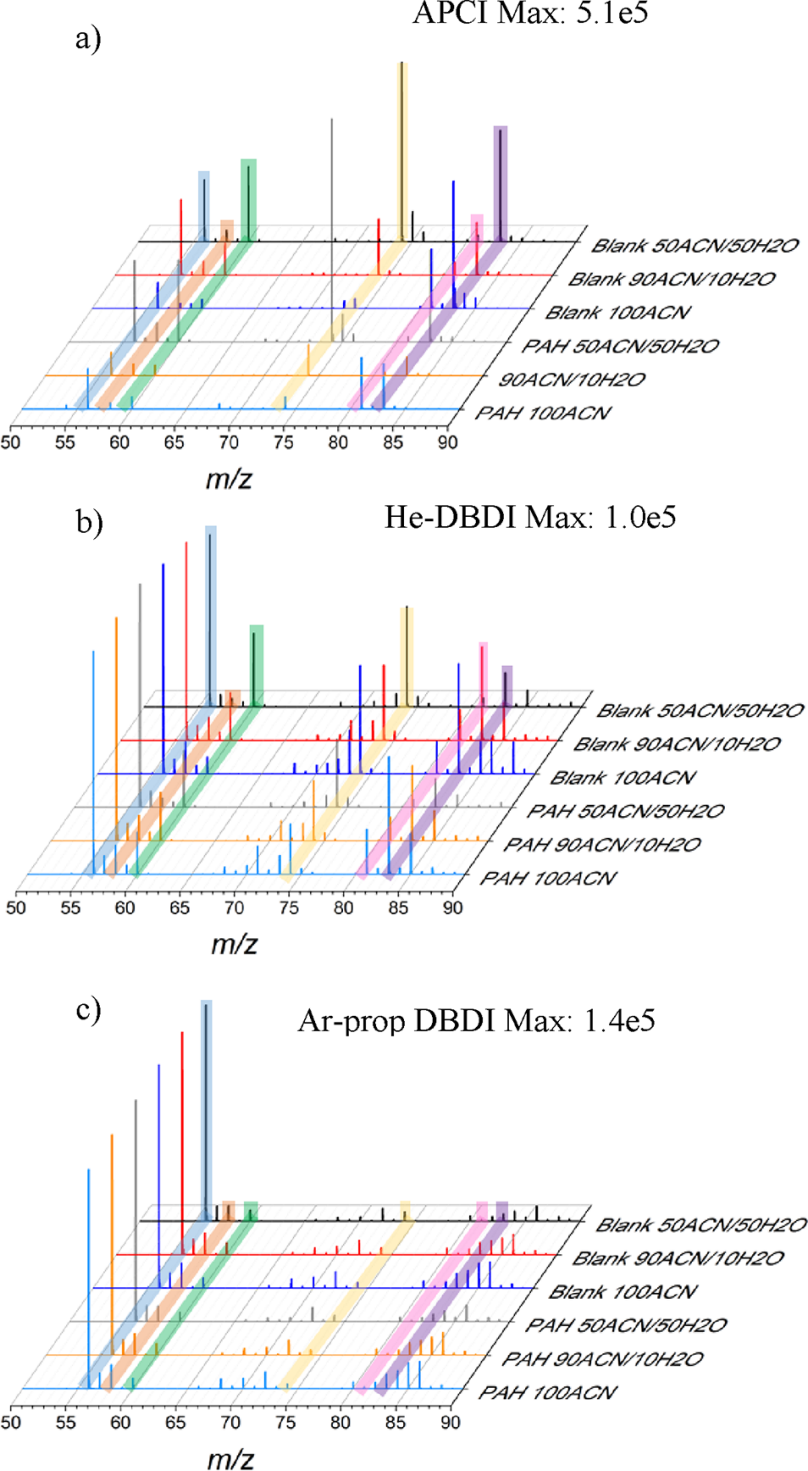
Reactant ions detected during LC-MS for the three ion source approaches used. (a) APCI, (b) He-DBDI, and (c) Ar-prop DBDI. The spectra of blank analysis, just chromatographic gradient, and a PAH analysis are collected at different solvent compositions. The PAH 90/10 data point corresponds with the peak for the B[*a*]ant + Chry elution. The main ion species detected and the potential molecular formula assigned are color coded in the figure. Blue label: *m*/*z* 56.0113 [N_4_]^·+^; orange label: *m*/*z* 58.0648 [C_3_H_7_N + H]^+^; green label: *m*/*z* 60.0442 [ACN + H_2_O + H]^+^; yellow label: *m*/*z* 74.0597 [C_3_H_7_NO + H]^+^, [DMF + H]^+^; pink label: *m*/*z* 81.0443 [C_4_H_5_N_2_]^+^; purple label: *m*/*z* 83.0599 [ACN_2_ + H]^+^. The intensities shown in a– c were normalized to the highest signal in the *m*/*z* interval (50–90) for each ion source approach.

In addition to N_4_^·+^, the background ions observed during the analysis can help to further understand the ruling ionization mechanisms. The region between *m*/*z* 50 and *m*/*z* 90 was data-rich for this type of ions (Figure 3). In the case of APCI, the major background species observed were also protonated ion species, as for PAHs. As shown in Figure 3a, the highest signal was observed for an easy-to-protonate molecule as *N*, *N*-dimethylformamide (DMF) when the mobile phase has 50% water (first minute of the chromatographic gradient). One of the primary sources of protons during APCI are the water clusters formed as reactant ions. The signal of [DMF + H]^+^ ion species at *m*/*z* 74.0597 was reduced when the amount of water was decreased during the LC gradient. Protonation was highly dependent on the presence of water clusters (see Figure 3a, Blank 50ACN/50H_2_O, Blank 90ACN/10H_2_O, and Blank 100ACN mass spectra). In addition, the co-solvent (ACN) has a higher PA than water but is lower compared to DMF. However, the high concentration of ACN can increase the probability of protonation and dimer formation (*m*/*z* 83.0599), promoting gas-phase competition during proton transfer reactions.

The presence of more abundant [M + H]^+^ species for PAHs analysis via APCI likely implies an environment with more proton donor species compared to He-DBDI. The PA of the proton donor species has a clear impact on the protonation of PAHs, observed by Williams et al. using a liquid sampling-atmospheric pressure glow discharge to analyze PAHs.^34^ In the present case, water cluster and ACN-protonated ions were detected as background ions (see Figure 3) in APCI and He-DBDI analyses. The PA values of the protonated PAHs can give us a hint of the influential water cluster formed. The PAHs detected as protonated species have PAs similar or higher than the second water cluster ([H(H_2_O)_2_]^+^) with a PA of 864.5 kJ/mol (see Table S2). If [H(H_2_O)]^+^ or [ACN + H]^+^ were abundant during APCI analysis of the gas phase, other PAHs should be detected as [M + H]^+^ (e.g., Flu with a PA of 832 kJ/mol).

The effect of PAH presence on the gas phase chemistry was evaluated by observing the changes in the background ions’ relative intensities. The results from blank (no sample injection) and PAH LC–MS analyses were compared. The N_4_^·+^ ion species showed a comparable intensity when the gradient was at 50/50 and 90/10 (ACN/water) and decreased when the mobile phase is just composed of ACN. The lower cutoff of the instrument used is *m*/*z* 50, but the radical ions of ACN (*m*/*z* 41.0260) cannot be detected to corroborate the potential charge exchange reaction with N_4_^·+^. On the other hand, when the PAHs were eluted (PAH 90/10, Figure 3a orange spectrum), N_4_^·+^ intensity experienced a signal decrease of 30% when compared to the blank (Figure 3a red trace). One of the potential reaction routes in APCI to form radical ion species are collected in eqs 3, 4, and 5 for both solvent and analytes, as the co-elution of B[*a*]ant + Chry. Corona discharge reactions seemed to consume N_4_^·+^ to form radical species (see Figure S1).

The shared plasma nature sometimes led to the wrong idea of assuming similar ionization mechanisms for different plasma-based ion sources. According to our results, DBDI had a different gas-phase chemistry when compared to APCI. First, more abundant signals for radical ions species were detected (Figure 2b), indicative of the predominant role of charge exchange reactions in He-DBDI plasma. Moreover, discharge gas Penning ionization reactions induce particular gas-phase chemistry going beyond plasma sustaining:
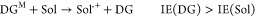
13
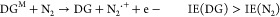
14

*DG^M^ corresponds to discharge gas metastable atoms.

As observed in Figure 3b,c, protonation in DBDI plasma ion sources was independent of the mobile phase composition changes suggesting a different reaction pathway. In the case of He-DBDI, the high IE (19.80 eV) of helium metastable (He^M^) atoms facilitated radical ion species formation by Penning ionization (eqs 13 and 14). The formation of water clusters (eq 6), the main protonation reactant ions in APCI, did not rely on nitrogen but on He^M^ atoms. Thus, no N_4_^·+^ signal decrease was observed during the chromatographic gradient. Still, the degree of protonation was smaller when compared to APCI.

Interestingly, a different discharge gas such as Ar-prop barely showed protonation for DBDI analysis. Argon metastable (Ar^M^) atoms have energies of 11.55 and 11.72 eV,^35^ but those energies are below N_2_ IE (15.6 eV); reactions 13 and 14 are highly unlikely. Still, N_4_^·+^ radical ion species were observed (Figure 3c). When compared to He plasmas where He and nitrogen ions are formed easily, Ar plasmas necessitate, mainly, from Ar ions to sustain the plasma and consequently from higher electron densities.^36^ The electron energy in Ar plasma should match or overcome argon’s IE (15.8 eV) to form Ar^·+^, and that energy is similar to N_2_ IE favoring eqs 1 and 2. Even when N_4_^·+^ was formed and detected, its intensity did not change during the gradient. Those ion species did not play a significant role in the Ar-Prop DBDI ionization mechanisms. The species that indeed can be involved in the gas-phase chemistry are propane molecules. In the argon–propane mixture, propane helps reduce the breakdown voltage and sustain the plasma by forming radical ion species; propane has a lower IE (10.94 eV) than Ar^M^ atoms.^37^

### Dopant-Assisted Ionization

**Figure 4:**
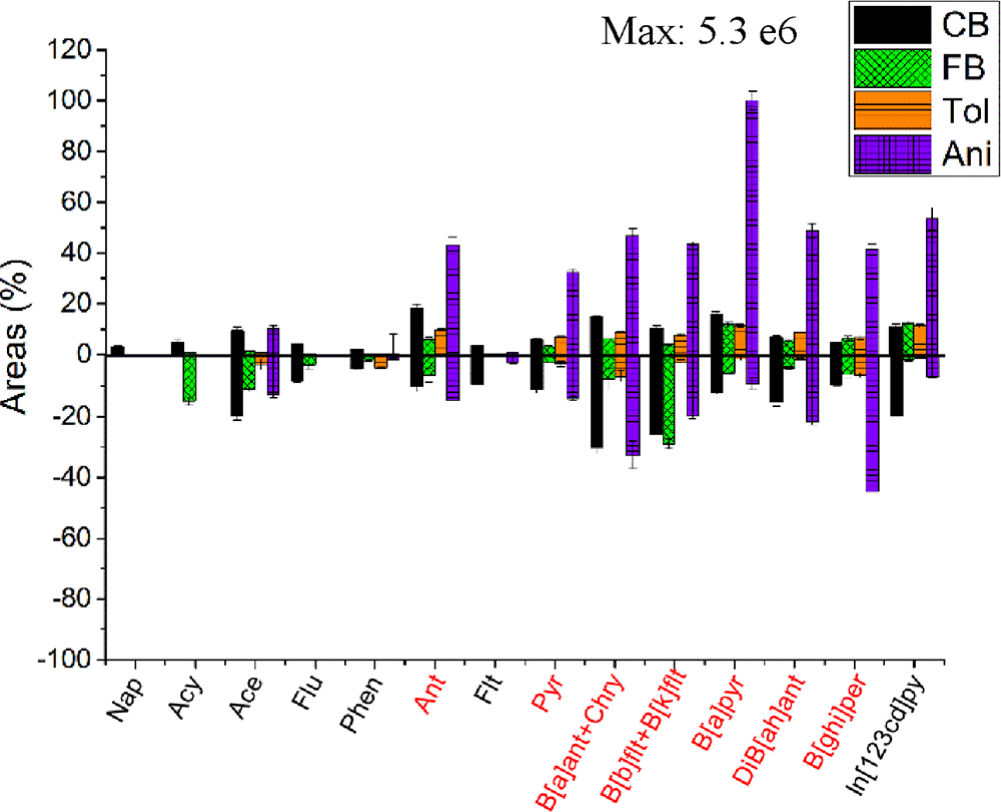
Effect of different dopants into the detection of PAH radical ion species. Positive values corresponds to He-DBDI analysis and negative values to APCI. To simplify the data interpretation, the PAHs’ peak areas were relativized to highest signals obtained in both analyses, [B[*a*]pyr]^·+^ intensity detected using He-DBDI. APCI data were adjusted to fit negative values and facilitate the figure interpretation; still, the intensities were not negative. Highlighted in red are the compounds with IE <7.70 eV.

The effect of organic dopants during ionization has been reported for different plasma-based ion sources.^27,38,39,43^ Four dopants (CB, FB, Tol, and Ani) with an IE range, covering from 8.20 to 9.20 eV, were used to evaluate the effect on our DBDI ionization mechanisms. The effect of the dopants for He-DBDI (upper part) and APCI (lower part) operation is shown in Figure 4. As expected, the radical ion species dominated the mass spectra, corroborating the trends observed in other plasma-based ion sources.^17,38^ Even in the case of APCI, PAH protonation was shifted toward charge exchange reactions (see Table S3).

Dopants were added in the gas phase to increase the charge-exchange reactions ionization efficiency and form radical ion species, similarly to atmospheric pressure photoionization (APPI).^40^ The selected dopants have an IE below Ar^M^ and He^M^ atoms; N_2_^·+^ and N_4_^·+^ IE should favor the formation of dopant radical ion species. However, the effect of each dopant was different.

CB and FB exhibited divergent behavior for APCI and DBDI. Radical ion species intensities were increased by the presence of CB in the three evaluated cases but with uneven efficiency (Table S3). APCI demonstrated the best performance with this dopant. A fold change over 2.2 compared to no dopant analysis was observed. Not only [PAH]^·+^ intensities were boosted, CB-related ion species showed increased intensities for APCI analysis. The better efficiency of the corona discharge ion source forming CB radical ion species was proven by the presence of ion species as [C_6_H_5_]^·+^ at *m*/*z* 77.0932, [C_6_H_5_^35^Cl]^·+^ at *m*/*z* 112.0081, and [C_6_H_5_^37^Cl]^·+^ at *m*/*z* 114.0074. Something similar was observed for FB when the corona discharge was the ion source.

CB and FB did not show a significant improvement in intensities for DBDI. These data should join Tol results, in which Tol was the dopant with less impact on the analytical signal. The ionization efficiency for most of the detected PAHs was diminished. Tol behavior can be explained by two different phenomena. During the LC–MS analysis, organic solvents such as methanol and ACN were used. Those solvents promoted a rapid reaction with the Tol radical ion species, similar to our case (see eq 15).^41,42^ Because ACN has a similar or higher PA than Tol, CB, and FB, the loss of a proton by the dopants likely inhibited further charge exchange reactions. Meanwhile, the PA of Ani PA (61 kJ/mol higher than ACN) should favor the dopant charge exchange reactions. In addition, Tol was fragmented during the analysis, especially for DBDI plasmas. Contaminants’ degradation to abate volatile organic compounds is a whole research topic in low-temperature plasmas.^43,44^ The highly reactive environment created by DBDI plasmas led to compound oxidation or fragmentation and, therefore, to side reactions with radicals that will reduce the probability of PAH interaction charge exchange reactions with Tol. Similar side reactions were observed for DBDI analysis using CB and FB as dopants (data not shown).
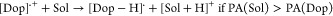
15

Dop means dopant.

The dopant that showed better results for radical ion species formation was Ani. The relatively low IE (8.20 eV) of Ani limited the opportunities for ionization via charge exchange reactions. Compounds with lower IE than Ani can be ionized (eq 10). Still, this is not the first report using Ani for plasma-based ionization,^38^ and in our case, it is the game changer in terms of sensitivity (Figure 4).

**Figure 5:**
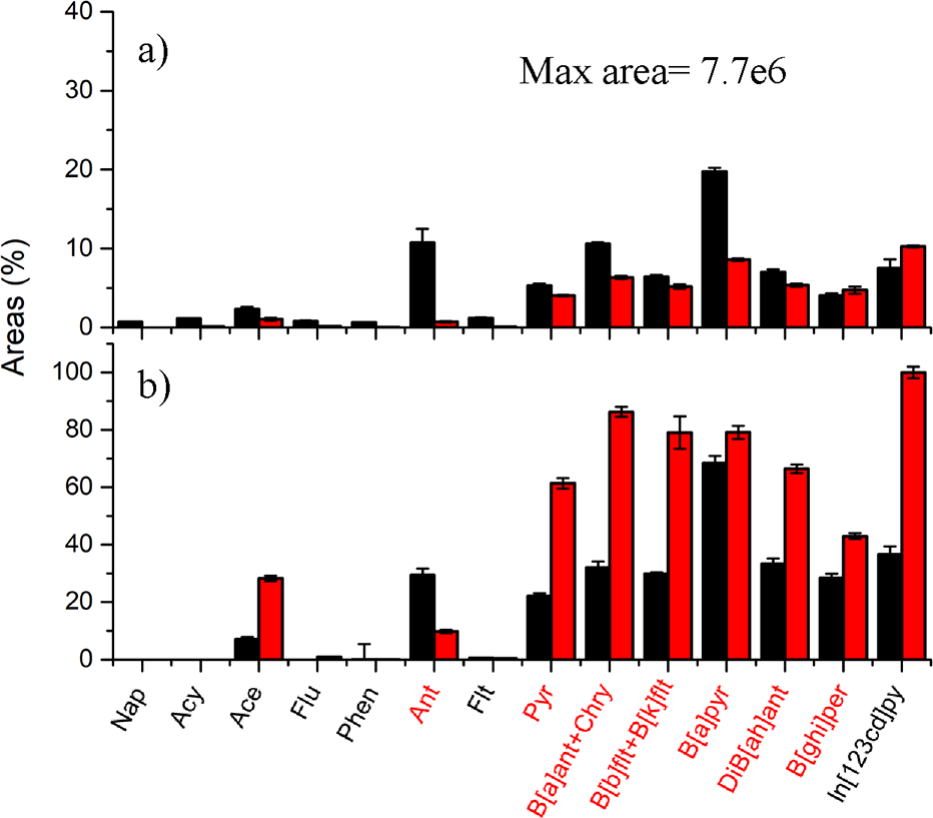
PAH radical ion species ([M]^·+^) intensities obtained using He (black bars) and Ar-propane (red bars) DBDI for (a) no dopant and (b) anisole. The intensities were relativized with respect to the maximum signal (In[123cd]py) Ar-prop+Anisole analysis. Highlighted in red are the compounds with IE <7.70 eV. To simplify the data interpretation, the PAHs’ peak areas were relativized to the highest signals obtained in both analyses, [In[123cd]py]·+ intensity detected using Ar-prop DBDI.

DBDI analysis using Ar-prop as discharge gas was less efficient in ionizing PAHs when compared to He-DBDI (Figure 5a). However, the ionization efficiency was drastically improved when Ani was combined with Ar-Prop-DBDI. The presence of the dopant in the gas phase boosted the intensities over nine times for most of the detected PAHs’ radical ion species (see Figure 5b). The ionization mechanism of the Ar-Prop mixture favored the presence of propane radical ion species ([C_3_H_8_]^·+^) in the plasma jet region.^45^ Those radical ion species likely boosted charge exchange reactions, not only for PAHs but also for dopants (see eq 10). We observed a higher probability of ionization when the dopant IE was lower: Ani = 8.20 eV < CB = 9.07 eV < FB = 9.20 eV. In the case of He-DBDI, the potential absence of He^M^ and similar highly energetic species in the plasma jet reduced the efficiency of Ani as a dopant to increase sensitivity. Four different calibration curves were performed to further compare the effect of Ani as PAH signals booster for Ar-prop, and He discharges (Figure S2). The argon discharge showed higher slopes, up to threefold steeper than He-DBDI, confirming the differences observed in Figure 5.

### Are Other Species Helping with Penning Ionization?

**Figure 6:**
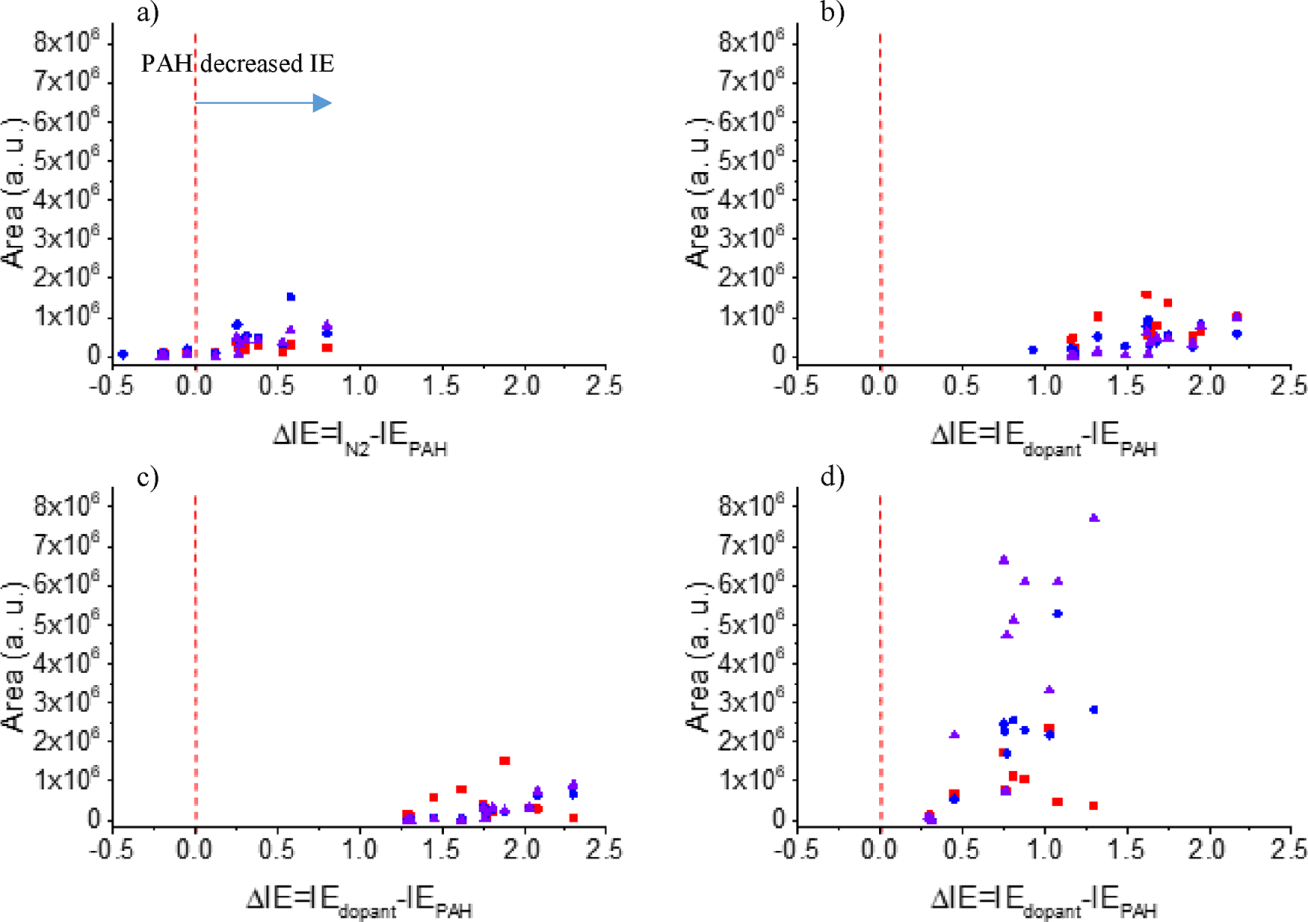
Areas of the different PAHs evaluated with respect to the total difference in between the IE of dopant and each PAH. Results for the [M]^·+^ detected for each PAH using as dopant. (a) No dopants (IE_N2B3Πg_ = 7.70 eV). (b) Chlorobenzene (IE_dopant_ = 9.07eV). (c) Fluorobenzene (IE_dopant_ = 9.20eV). d) Anisole (IE_dopant_ = 8.20eV). Red squares corresponded to APCI results, blue diamonds for He-DBDI results, and purple triangles for Ar-Prop-DBDI results.

In theory, the high IE of He^M^ is enough to ionize the evaluated molecules. However, the short lifetime of the metastable atoms in the open atmosphere and the low intensities for PAHs with IE higher than 7.7 eV suggested otherwise (Figure 6a). The participation of other excited species, easily formed and long living in the plasma jet region, feels mandatory to ionize the PAHs.

The IE difference between the excited molecule and the target analyte for charge exchange reactions is a critical parameter. Like in photoionization, the energy transfer is more efficient when the energy of the ionization system is closer to the analyte IE (IE_excitation_ ≈ IE_analyte_). With this in mind, the parameter ΔIE (ΔIE = IE_dopant/N2B3Πg_ – IE_PAH_) was proposed to highlight the importance of an efficient energy transfer during the reaction. The general assumption is that if the dopant is ionized and has a higher IE than the analyte, [M]^·+^ intensity should be boosted. FB (9.20 eV) and CB (9.07 eV) partially corroborated that for APCI analysis (red squares in Figure 6b,c). Meanwhile, for DBDI, the species with lower ΔIE had higher intensities, but the increment was not that significant (Table S3).

Therefore, what if other species are contributing to Penning ionization, especially for DBDI? Among the PAHs evaluated, there is a group of them with IE <7.70 eV, shadowed in Figure 1 or highlighted with red font in Figures 2, 4, and 5. Traditionally, nitrogen and APCI are linked. Beyond other potential ionization mechanisms, if nitrogen ions were formed in the plasma, independently of the region, there must be species (atoms or electrons) capable of matching the 15.6 eV nitrogen IE. At the same time, that energy is high enough to form other nitrogen-related species, such as the N_2_ C^3^Π_u_ intermediate excited state. This excited state requires around 11 eV to be formed, easily matched by He^M^ or Ar^M^. In the past, the same DBDI plasmas as the one used in this work showed emission lines for this transition, at 337 nm (0,0), 357 nm (0,1), and 380 nm (0,2), for He-DBDI and Ar-prop-DBDI operation.^46,45^ However, the Ar-prop excited species in the DBDI plasma jet did not have enough energy to promote N_2_^+^ or N_2_ B^2^∑_u_^+^ species and no emission line was observed at 391 nm.^45^ The internal transition from N_2_ C^3^Π_u_ to the lower vibrational state N_2_ B^3^Π_g_ under those conditions is possible. The addition of the ground state energy of N_2_ B^3^Π_g_ (7.30 eV) and the energy gap between 337 and 380 nm lines (∼0.4 eV) matched the 7.70 eV threshold observed for the PAHs. The high population of nitrogen, both as discharge gas impurity and as part of the ambient atmosphere, makes N_2_ a potential candidate for auxiliary Penning ionization.

This system had been previously proposed as potential Penning ionization for other ambient ion sources.^25,47^ In the absence of other species, N_2_ B^3^Π_g_ excited molecules can be held accountable for the formation of radical ion species. Our results are in agreement with that hypothesis.

The same behavior was observed for Ani but promoted more efficient ionization (see Figure 6d). Ani is the dopant with the lowest IE (8.20 eV), close to the energy threshold for N_2_ B^3^Π_g_ molecules and right next to the IE of the last PAH under study (IE_Nap_ = 8.14 eV). The observed data implies a scenario where charge exchange reactions require slightly higher IE of atoms or molecules transferring the charge compared to the analyte’s IE for more efficient energy transfer and therefore radical ion species formation.

## Conclusions

Plasma-based ionization mechanisms have long been considered similar to APCI. However, we observed substantial differences in ionization mechanisms ruling vaporized liquid analysis using APCI and DBDI. Protonated and radical ion species showed different trends for each ion source, even when analyzed under the same conditions. Corona discharge ionization mechanisms, as expected, relayed on N_2_ reactions and the solvent composition to control the ionization mechanisms. The ion N_4_^·+^ was detected and seemed to play a role not only during the formation of water clusters and protonation but also during APCI charge exchange reactions to form radical ion species of PAHs. In contrast, DBDI ionization was less influenced by the solvent composition and solely relayed in the discharge gas and the atmosphere components.

The ionization mechanisms were shifted toward charge exchange reactions by using dopants. The results showed greater efficiencies of DBDI discharges for the detection of [M]^·+^. Among the evaluated dopants, Ani displayed the best results, boosting over three times for most of the compounds detected with the three approaches evaluated. The results obtained in the present work suggested the presence of side, overlooked reactions as potential ionization mechanisms. Even when higher energy helium metastable atoms were present in the plasma, we cannot effectively ionize all the PAHs under study. Other reactive species, such as N_2_ B^3^Π_g_ nitrogen excited state or Ani, apparently played a key role in low IE (IE ≤ 7.70 eV) molecule ionization for both APCI and DBDI plasmas.

The gained understanding of the ionization mechanisms for both APCI and DBDI will permit future work to deepen and improve LC–MS and gas chromatography-MS plasma-based analysis performance, with special emphasis on radical ion species analysis. The effect of LC-related parameters as the mobile phase composition or the flow rate should be considered in future studies.

## Supporting Information Available

Detailed information and experimental results, including the formula, molecular weight, IE, and PA of most of the compounds used in the experiments; the proton affinities of the main water clusters (*n* ≤ 5); the radical and protonated ion species ratios for most of the analysis in the manuscript; the evolution of the N_4_^·+^ signal during APCI analysis; calibration curves for B[*a*]pyr and B[*b*]flt for DBDI analysis with different discharge gases using anisole as dopant; and the effect of anisole in high PA compound protonation for He-DBDI and Ar-Prop-DBDI (PDF)
