# Supplementary material for: Dielectric Barrier Discharge Ionization Mechanisms: Polycyclic Aromatic Hydrocarbons as a Case of Study
Source: Anal Chem. 2022 Dec 20;95(2):854–61. doi: 10.1021/acs.analchem.2c03279 (PMC9850405; doi:10.1021/acs.analchem.2c03279)
Supplement: Supplementary file 1 — ac2c03279_si_001.pdf [file ac2c03279_si_001.pdf]

Supporting information

Dielectric barrier discharge ionization mechanisms: polycyclic aromatic hydrocarbons as a case of study

Marcos Bouza*[1], Julio García-Martínez[1], Bienvenida Gilbert-López[1], Sebastian Brandt[2], Juan F. García-Reyes[1], Antonio Molina-Díaz[1] and Joachim Franzke*[2]

[1]Analytical Chemistry Research Group, Department of Physical and Analytical Chemistry, University of Jaén, Campus Las Lagunillas, 23071 Jaén, Spain.

[2]ISAS—Leibniz Institut für Analytische Wissenschaften, Bunsen-Kirchhoff-Str. 11, 44139 Dortmund, Germany.

*Corresponding authors E-mail: Marcos Bouza: mbouza@ujaen.es, and Joachim Franzke: franzke@isas.de.

Table of Contents

Table S1. Compounds used for the different studies. The abbreviation, formula, molecular weight, ionization potential (eV) and proton affinity (kJ/mol), when available, are collected..**S3**

Table S2. Proton affinities of different water clusters. Data obtained from reference 5 and NIST Chemistry webbook website (https://webbook.nist.gov/chemistry/) (*)......................**S5**

Table S3. Ratio of the [M]·+ and [M+H]+ intensities for the signals of He-DBDI and Ar-Prop-DBDI with respect to corona discharge (APCI). In addition, the ratios of signals for different PAH ions species when a dopant is used with respect to the signal without any dopant are collected. The orange columns corresponds to APCI, blue columns to He-DBDI and the purple ones to Ar-Prop-DBDI..**S6**

Figure S1. Temporal evolution of the $N_4^{\cdot+}$ (*m/z*=56.0113) signal and the *m/z* 228.0934 during the LC-MS PAH analysis using APCI as ion source. a) 1 min run, b) starting of B[a]ant+Chrys chromatographic peak, c) B[a]ant+Chrys chromatographic average, d) after compounds elution, e) 10 min run. The red dashed line corresponded to $N_4^{\cdot+}$ intensity when the PAH eluted. $N_4^{\cdot+}$ signal suffer a reduction when the Ban[a]ant and Chry were in the gas-phase and after the compounds eluted the signal increased again...**S7**

Figure S2. Calibration curves built using concentrations from 250 ppb to 5000 ppb and anisole as dopant for: a) B[a]pyr, and b) B[b]flt. The red traces correspond to Ar-prop-DBDI signals and the black traces to He-DBDI..**S8**

Table S1. Compounds used for the different studies. The abbreviation, formula, molecular weight, ionization potential (eV) and proton affinity (kJ/mol), when available, are collected.

Compound	Abbreviation	Formula	*Molecular weight*	Ionization Potential (eV)	Proton affinity (kJ/mol)
Naphthalene	Nap	$C_{10}H_8$	128.0626	8.14*	803*
Acenaphthylene	Acy	$C_{12}H_8$	152.0626	8.02*	861*
Acenaphtene	Ace	$C_{12}H_{10}$	154.0783	7.75*	852*
Fluorene	Flu	$C_{13}H_{10}$	166.0783	7.91*	832*
Phenanthrene	Phen	$C_{14}H_{10}$	178.0783	7.89*	826*
Anthracene	Ant	$C_{14}H_{10}$	178.0783	7.44*	877*
Fluoranthene	Flt	$C_{16}H_{10}$	202.0783	7.90*	829*
Pyrene	Pyr	$C_{16}H_{10}$	202.0783	7.43*	869*
Benzo[a]anthracene	B[a]ant	$C_{18}H_{12}$	228.0934	7.45*	869*
Chrysene	Chry	$C_{18}H_{12}$	228.0934	7.60*	841*
Benzo[b]fluoranthene	B[b]flt	$C_{20}H_{12}$	252.0939	7.32[1]	-
Benzo[k]fluoranthene	B[k]flt	$C_{20}H_{12}$	252.0939	6.99[1]	856[2]
Benzo[a]pyrene	B[a]pyr	$C_{20}H_{12}$	252.0939	7.12*	890[2]
Dibenz[a,h]anthracene	DiB[ah]ant	$C_{22}H_{14}$	278.1096	7.39*	856[2]
Benzo[g,h,i]perylene	B[ghi]per	$C_{22}H_{12}$	276.0939	7.17*	876*
Indeno[1,2,3-cd] pyrene	In[123cd]py	$C_{22}H_{12}$	276.0939	6.90[1]	866[2]
Chlorobenzene	CB	C_6H_5Cl	112.0080	9.07*	753*
	[CB-H]$^{\cdot}$	C_6H_4Cl	110.9996	-	913*
Fluorobenzene	FB	C_6H_5F	96.0375	9.20*	756*
	[FB-H]$^{\cdot}$	C_6H_4F	95.0292	-	900*
Toluene	Tol	C_7H_8	92.0626	8.83*	784*
	[Tol-H]$^{\cdot}$	C_7H_7	91.0542	-	838*
Anisole	Ani	C_7H_8O	108.0575	8.20*	840*

	[Ani-H]$^{\cdot}$	C_7H_7O	107.0491	-	995*
Acetonitrile	ACN	C_2H_3N	41.0265	12.20*	779*
	[ACN-H]$^{\cdot}$	C_2H_3N	40.0182		538[3]
Water	H_2O	H_2O	18.0106	12.621*	691*
	[H_2O-H]$^{\cdot}$	HO	17.0022	-	593[3]
Dimethyl formamide	DFM	C_3H_7NO	73.0528	9.13*	887.5*
Propane	Prop	C_3H_8	44.0626	10.94*	626*
	[Pro-H]$^{\cdot}$	[C_3H_7]$^{\cdot}$	43.0542	-	671[4]

Values obtained from NIST Chemistry webbook website (https://webbook.nist.gov/chemistry/) (*), and references 1-4.

Table S2. Proton affinities of different water clusters. Data obtained from reference 5 and NIST Chemistry webbook website (https://webbook.nist.gov/chemistry/) (*).

Cluster [H(H2O)n]+, n	Proton affinity (kJ/mol)
1	725.3[5] (691*)
2	864.5[5]
3	893.45[5]
4	935.91[5]
5	964.85[5]

Table S3. Ratio of the $[M]^{\cdot+}$ and $[M+H]^{+}$ intensities for the signals of He-DBDI and Ar-Prop-DBDI with respect to corona discharge (APCI). In addition, the ratios of signals for different PAH ions species when a dopant is used with respect to the signal without any dopant are collected. The orange columns corresponds to APCI, blue columns to He-DBDI and the purple ones to Ar-Prop-DBDI.

$[M]^{\cdot+}$	He-DBDI/APCI	Ar-Prop-DBDI/APCI	CB/ND	FB/ND	Tol/ND	Ani/ND	CB/ND	FB/ND	Tol/ND	Ani/ND	CB/ND	FB/ND	Tol/ND	Ani/ND
**Nap (8.14 eV)**	--	--	--	--	--	--	3.2	--	--	--	--	--	--	--
**Acy (7.58 eV)**	0.78	0.08	--	7.0	--	--	2.9	0.6	--	--	4.4	1.0	0.7	--
**Ace (7.75 eV)**	1.90	0.85	10.9	6.0	1.5	7.1	2.8	0.4	0.3	3.0	1.5	0.4	0.9	26.9
**Flu (7.91 eV)**	0.86	0.19	5.8	2.2	--	--	3.4	0.4	--	--	1.6	0.7	0.7	5.3
**Phen (7.89 eV)**	0.55	0.03	2.5	0.9	2.3	0.7	2.2	0.2	0.2	**0.1**	1.5	0.4	0.3	3.5
**Ant (7.44 eV)**	4.05	0.26	2.6	1.6	--	3.8	1.2	0.4	0.6	2.7	1.5	0.7	0.9	13.9
**Flt (7.90 eV)**	0.94	0.06	5.0	--	--	1.3	2.0	0.2	0.2	0.4	1.6	0.5	0.7	5.4
**Pyr (7.43 eV)**	2.28	1.75	3.3	0.6	0.8	4.1	0.7	0.4	0.9	4.2	1.2	0.6	1.0	15.1
**B(a)ant + Chry (7.45/7.60 eV)**	2.35	1.41	4.6	1.2	1.0	5.0	1.0	0.4	0.6	3.0	1.3	0.7	1.0	13.6
**B(b)flt + B(k)flt (7.32/6.99 eV)**	1.80	1.45	5.0	5.6	0.4	3.8	1.1	0.4	0.8	4.7	1.2	0.6	0.9	15.3
**B(a)pyr (7.12 eV)**	5.18	2.25	2.2	1.0	0.2	1.6	0.5	0.4	0.4	3.5	1.2	1.1	0.9	9.2
**DiB(ah)ant (7.39 eV)**	2.93	2.25	4.4	1.2	0.3	6.2	0.7	0.5	0.8	4.8	1.2	0.8	1.0	12.4
**B(ghi)per (7.17 eV)**	2.17	2.54	3.5	2.2	2.3	16.4	0.8	1.1	1.1	7.0	0.9	0.8	1.1	9.1
**In(123cd)py (6.90 eV)**	2.54	3.45	4.5	0.3	0.2	1.6	1.0	1.1	1.0	4.8	1.3	1.1	0.9	9.8
$[M+H]^{+}$	**He-DBDI/APCI**	**Ar-Prop-DBDI/APCI**	**CB/ND**	**FB/ND**	**Tol/ND**	**Ani/ND**	**CB/ND**	**FB/ND**	**Tol/ND**	**Ani/ND**	**CB/ND**	**FB/ND**	**Tol/ND**	**Ani/ND**
**Nap (803 kJ/mol)**	0.19	--	--	--	--	--	2.4	--	--	--	1.5	1.1	1.4	0.5
**Acy (861 kJ/mol)**	--	0.0	1.4	0.1	0.3	--	0.1	1.2	--	--	4.1	0.7	0.6	--
**Ace (852 kJ/mol)**	0.43	2.73	4.7	12.4	7.3	17.8	0.3	0.3	0.4	1.8	0.1	0.3	0.2	1.0
**Flu (832 kJ/mol)**	1.39	0.90	0.6	2.9	1.1	2.7	0.9	0.3	--	--	1.9	0.5	0.7	3.3
**Phen (826 kJ/mol)**	0.25	--	--	3.1	9.3	4.9	0.7	0.6	0.7	1.0	1.2	0.7	1.0	16.7
**Ant (877 kJ/mol)**	0.37	0.06	0.1	0.1	0.1	--	0.4	0.4	0.2	--	1.8	--	--	3.3
**Flt (829 kJ/mol)**	0.04	0.37	--	--	--	13.1	--	7.6	2.2	13.3	1.0	0.9	1.1	--
**Pyr (869 kJ/mol)**	0.03	--	--	--	--	0.3	0.5	0.9	0.9	--	4.5	33.7	--	--
**B(a)ant + Chry (869/841 kJ/mol)**	--	--	--	--	--	0.1	1.1	1.5	1.1	1.2	1.5	1.3	1.6	10.9
**B(b)flt + B(k)flt (856/– kJ/mol)**	0.03	0.11	0.3	--	--	1.6	--	--	--	--	0.7	--	0.9	--
**B(a)pyr (890 kJ/mol)**	0.02	0.01	--	--	--	--	0.5	0.3	0.7	--	0.9	0.2	0.9	--
**DiB(ah)ant (856 kJ/mol)**	0.04	--	--	--	--	--	0.9	0.3	1.2	--	--	--	--	--
**B(ghi)per (876 kJ/mol)**	0.02	--	--	--	0.1	--	0.6	0.2	0.6	0.9	--	--	--	--
**In(123cd)py (866 kJ/mol)**	0.19	--	--	--	--	--	0.8	0.2	0.8	0.8	--	2.2	0.2	4.5

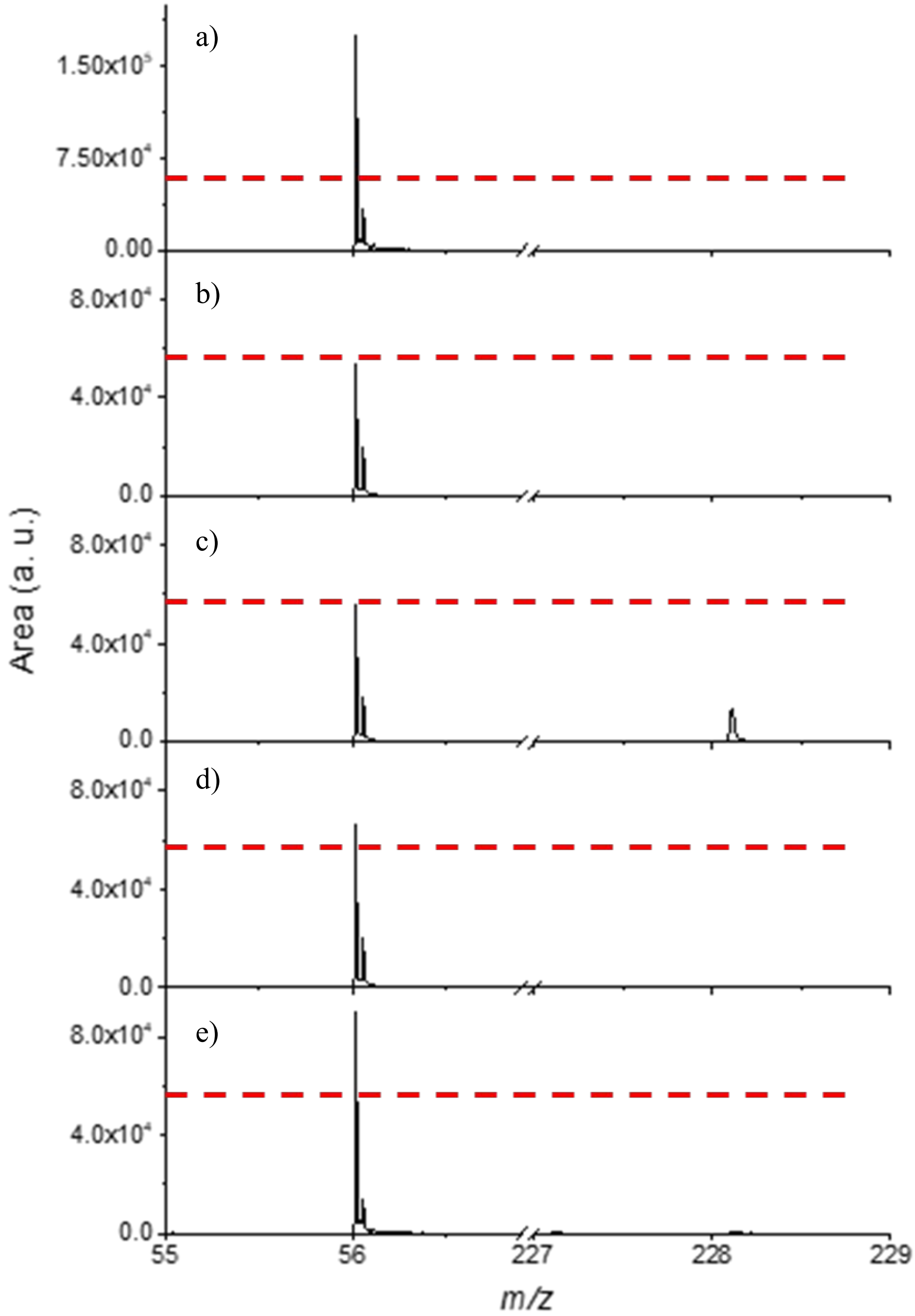

Figure S2. Temporal evolution of the $N_4^{\cdot+}$ (*m/z*=56.0113) signal and the *m/z* 228.0934 during the LC-MS PAH analysis using APCI as ion source. a) 1 min run, b) starting of B[a]ant+Chrys chromatographic peak, c) B[a]ant+Chrys chromatographic average, d) after compounds elution, e) 10 min run. The red dashed line corresponded to $N_4^{\cdot+}$ intensity when the PAH eluted. $N_4^{\cdot+}$ signal suffer a reduction when the Ban[a]ant and Chry were in the gas-phase and after the compounds eluted the signal increased again.

a)

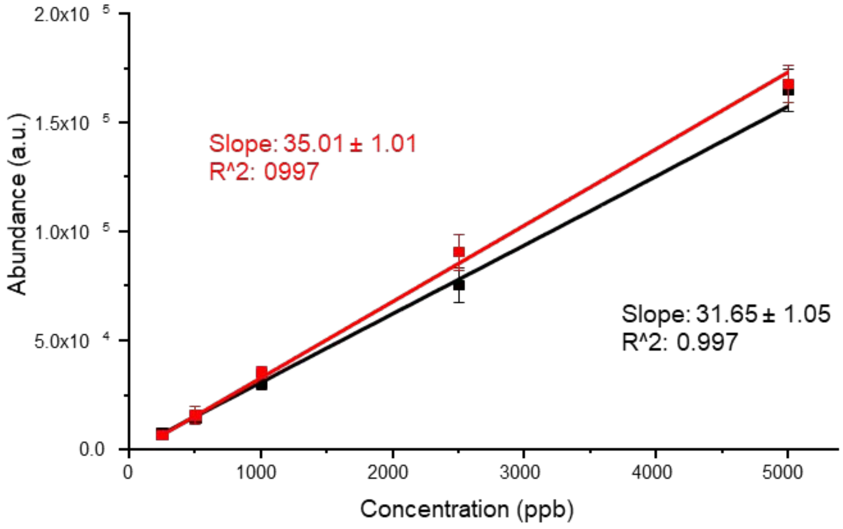

b)

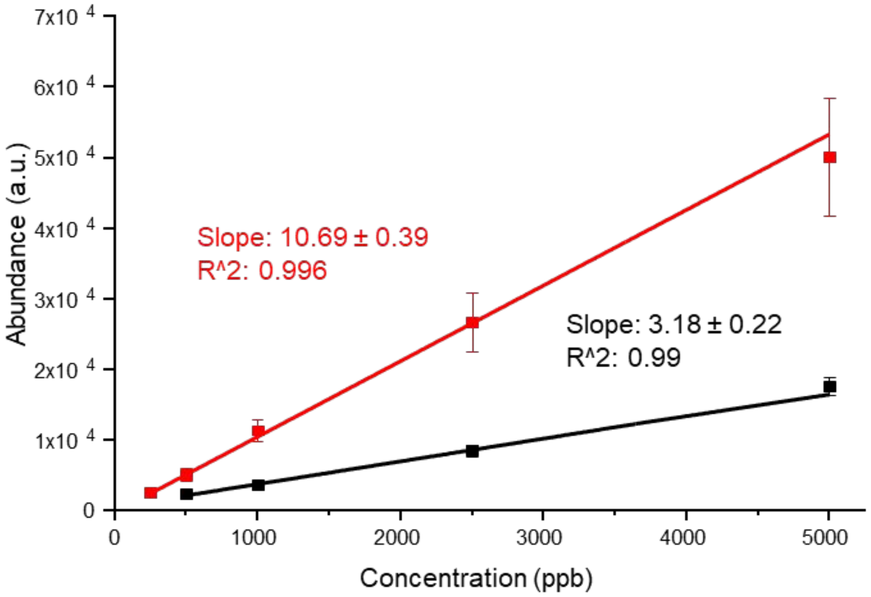

Figure S2. Calibration curves built using concentrations from 250 ppb(ng/mL) to 5000 ppb(ng/mL) and anisole as dopant for: a) B[a]pyr, and b) B[b]flt. The red traces correspond to Ar-prop-DBDI signals and the black traces to He-DBDI.

References

[1]Gutierrez-Urbano, I.; Villen-Guzman, M.;Perez-Recuerda, R.;Rodriguez-Maroto, J. M. Removal of polycyclic aromatic hydrocarbons (PAHs) in conventional drinking water treatment processes, *J. Contam. Hydrol.* **2021**, *243,* 103888.

[2]Aue, D. H.; Guidoni, M.; Betowski, L. D. Ab initio calculated gas-phase basicities of polynuclear aromatic hydrocarbons, *Int. J. Mass Spectrom.* **2000**, *201*, 283-295.

[3]Song, L.; Gibson, S. C.; Bhandari, D.; Cook, K. D.; Bartmess, J. E. Ionization Mechanism of Positive-Ion Direct Analysis in Real Time: A Transient Microenvironment Concept. *Anal. Chem.* **2009**, *81 (24),* 10080-10088.

[4]Hunter, E. D. L.; Lias, S. G. Evaluated Gas Phase Basicities and Proton Affinities of Molecules: An Update*, J. Phys. Chem. Ref. Data* **1998,** *27*, 413-656.

[5]H. Cheng. Water Clusters: Fascinating Hydrogen-Bonding Networks, Solvation Shell Structures, and Proton Motion, *J. Phys. Chem. A* **1998**, *102*, 6201–6204.
